# Veratrum Album*In consequence of a misunderstanding, the second half of this lecture was printed first—in our October number. Dr. Kent having notified us of the blunder and remitted us the missing first part, it is accordingly published.—Eds.

**Published:** 1887-03

**Authors:** J. T. Kent


### VERATRUM ALBUM.*

A LECTURE BY PROFESSOR J. T. KENT, A. M., M. D.

[Stenographically Reported.]

This is the white hellebore, the root being the part used, prepared by tincture in the usual way. Veratrum alb. is a friend of the woman; it is pre-eminently the woman's remedy; it corresponds to many complaints in them not at all found in men. Especially is it mixed up with the hysterical diathesis. Veratrum alb. develops many functional uterine disturbances; develops the mental states with it; hence, in mental complaints, associated with uterine disturbances, it is of great value.

Hahnemann made the statement that the lunatic asylums would be diminished by one-third if these insane doctors, or doctors that treat crazy folks, were familiar with the use of Veratrum alb. He meant that it would cure one-third of the female lunatics in the asylums of his day. Of course, insanity is treated much better now than it was then, and perhaps his remark would not apply. Nevertheless, it is frequently indicated in the insane freaks and mental wrongs of women. It may also be used in men, but not so frequently. You will

---

*In consequence of a misunderstanding, the second half of this lecture was printed first—in our October number. Dr. Kent having notified us of the blunder and remitted us the missing first part, it is accordingly published.—EDS.

notice running through the remedy that the mental states are all made worse at the menstrual epoch. It has many symptoms related to the menstrual period: hysterical freaks of a nonsensical character; vomiting, diarrhœa, and headaches coming on at the menstrual period. Now you see the general aggravation. Nearly all the acute exacerbations of the complaints running through Veratrum alb. are marked by vomiting, diarrhœa, and sweat. The sweat is all over the body and it is cold, especially upon the face and head; profuse sweat; and with all this there is great exhaustion. This runs through the acute, sharp, striking complaints produced by this remedy. There is a copiousness marking all these features—copious vomit, copious diarrhœa, and copious sweat. Arsenicum is sometimes distinguished from Veratrum alb. because of the scantiness of all these things, of all these peculiar symptoms. In Arsenicum we have scanty discharges; true, we have there the exhaustion, the sweat, and the vomit; but there is more retching, frequently vomiting up a little at a time, while in Veratrum alb. it is copious. The sweat is not so profuse in Arsenicum. In Veratrum alb. it is as cold as ice; it seems cold when it comes out. In Arsenicum it comes out warm on the body, and becomes cold afterward. Arsenicum has frequent, scanty discharges from the bowels, but they are generally scanty—they are not copious. In Veratrum alb. they are copious. Both have marked exhaustion; both have restlessness; both have pain and symptoms of restlessness driving them to despair, and driving them out of bed; and both have aggravations in the night. This remedy has burning running through it like Arsenicum, but it is distinguished by the copiousness of the excretions and secretions. Burning in the brain, burning in the stomach, burning in the abdomen, are characteristic of Veratrum alb. But we have all these burnings in Arsenicum also, and you must think of Arsenicum first of any remedy. Veratrum alb. has thirst for cold drinks, like Arsenicum, little and often. Now, see how easy it is to get two remedies mixed up when you only take a part of the picture of either. Veratrum alb. has an appetite for cold things, cold food, fruits, juices, succulent things, refreshing things, and is aggravated by warm things and warm drinks. Arsenicum is made better by warm things and warm drinks. There we have its opposite. Coldness in various modifications you will see running through the remedy. There is a sensation in Veratrum alb. as if a lump of ice were lying on the head, it is so cold—icy cold. There is coldness in the stomach, coldness in the abdomen at times, but this is circumscribed in its locality; it is in keeping with the general picture, the general state. The hands and feet are cold as ice with or without sweating; there is marked general bodily coldness; coldness all over the body, occurring usually with a profuse sweat.

Now, with this coldness, blueness is common. It is not the congestive blueness of Pulsatilla nor of Lachesis, but rather a pale, bluish appearance from relaxed blood-vessels; blueness of the hands. You will need to know Veratrum alb. in this blueness. The veins of the hands and feet fill up and appear full; by pressure they become pale, and it is a long time before the pale part becomes changed to its original blueness again. The blueness disappears under pressure and remains away quite a while. This blueness is peculiar to Veratrum alb., blueness of the surface; it may exist anywhere upon the surface; blueness of the face, blueness of the skin, blueness of the hands, blueness of the feet. You will see this condition after sudden exhaustion, after great vomiting, after great diarrhœa; vomiting, diarrhœa, and profuse sweat. Sometimes when this bluish condition of the skin appears the ears will be almost transparent with cold; the nose, and especially the tip of the nose, will be cold and elongated apparently, because it is shrunken or shriveled, looking more pointed; the general appearance of the face is more pointed; there is deathly sickness; icy coldness of the fingers and toes, and in cavities. Such a state pre-eminently belongs to Veratrum alb. The pains generally come on in the night, and commonly in the latter part of the night; relief only comes by walking. The patient will tumble around in the bed, but that will not relieve the pains; they drive to despair, and so the patient gets up and walks about for relief. Even in rheumatism where there is much soreness and swelling of the joints and tenderness, yet the excruciating pains make him forget the soreness so that he will get up and walk around in the night. (Mercurial pains are worse from the warmth of the bed and are relieved from cold.) Veratrum alb. has a sudden sinking of strength, sudden prostration, like Arsenicum, Camphor, and Secale. There is one general condition that belongs to many of the complaints of Veratrum alb.: external coldness with internal heat; associated with this we have dark, cloudy urine. There are three grand characteristics combining that distinguish Veratrum alb. from other remedies: the surface of the body is cold to the touch—as cold as death—he may be covered with sweat, cold sweat, even shaking; great chilliness, shuddering; hands and feet icy cold with a sensation of great heat; with great thirst; dryness of the mouth, tongue, and palate; sense of inner burning; burning in the stomach; burning in the brain; burning in the abdomen; and dark-colored urine.

That is very characteristic of Veratrum alb. In the beginning I told you that Veratrum alb. corresponds in its mind symptoms with many of the wrongs of the insane. It has acute delirium, and many of the mind symptoms are of a sort of quasi-hysterical character. The insane person or woman desires to kiss everybody. This symptom runs through the text to a great extent. During her menstrual period she becomes hysterical and wants to kiss everybody. When you find that symptom in the sick-room you won't laugh at it. you will want a remedy to get rid of it, or you will be turned out of doors. No good husband is going to watch you tampering around with your nonsensical doses of medicine unless you relieve his wife from that symptom; he doesn't believe in nonsense. These things are not told you to laugh at. I tell them to you in dead earnest. If you think I am joking about it, wait until you get to the sick-room, and see how you succeed. Now, like Anacardium, there is a tendency to curse and swear; even a devout and pious woman in her hysterical freaks will become profane and curse and swear; but this remedy has not the two wills of Anacardium; it is more active in character. It also has religious melancholy, and despair of her position in society; that is a marked feature, a very marked feature. The woman imagines she has done something whereby she has forfeited her position among her friends and in society, and so takes on about it. Despair of salvation or despair of her position in society may come on with suppressed menses, with profuse menses, or when too soon, or any menstrual disorder; it may come on at the climacteric; but you will find a phase of it that is associated with some menstrual disorder. These symptoms that appear to correspond to Anacardium are entirely different, because the Anacardium symptoms are in no way related to the uterine complaints; they properly belong to the head and brain. Veratrum alb. has very little relation to the brain. It has mental disorders coming on in consequence of injured pride or fright. Some women have a superabundance of pride; they often become haughty, nonsensically so, and the slightest thing seems to trample upon their wonderfully false notions. The haughtiness breaks them down. The haughtiness itself may be a disease—may be a wrong state of the body, and Platina very often corresponds to it. All of the complete symptoms running through the text will have some portion of the red string attached to it.

Headache: head hot and covered with sweat; cannot bear to be left alone. That runs through many of the symptoms of this remedy; cannot bear to be left alone. With the headache there is nausea, vomiting, paleness, and stiff neck; micturition; pains coming on in the afternoon last through the night. Drawing in both arms; burning pains drive him to despair; with great prostration; with headache; with fainting; with cold sweat, great thirst, nausea, vomiting, and diarrhœa. You get in that not only the headache, but the headache with its peculiarities, with the vomiting, diarrhœa, and the great sweat. Perhaps it is associated in some way with menstrual disorders, with the wrongs that are likely to occur in menstruation, or at the time of menstruation. In these symptoms it stands out, carrying with it all its peculiarities, especially in the symptom: burning pains drive to despair; great prostration with the headache, with cold sweat; with great thirst, nausea, vomiting, and diarrhœa. The following is not a very important symptom, but Veratrum alb. has contraction of the pupils and also its opposite. Some patients experience this when they first take the remedy, when, by some authors, it is called the primary effect, and get the secondary symptom later on. Another individual will take it and reverse the order of things, so that which is a primary symptom in one is a secondary symptom in another. You must not get confused as to the homœopathic primary and secondary symptom. If your remedy fits the symptom it will cure it regardless of whether it comes from its primary or secondary effect.

In Veratrum alb. there is a restless, wild look, and a distortion of the face. In collapse it is related to Camphor and Arsenicum; we have collapse, bluish appearance of the face, nose more pointed, of a reddened hue; that is, if it is red in bed it will become pale on rising—alternately pale and red; drawing, tearing pains, with bluish, pale face, sunken eyes, and prostration. We have coldness in Camphor; it is, in fact, quite characteristic of Camphor to be icy cold without any sweat; the tongue is actually cold, withered, and swollen; dry, cracked and red; white with red tips, and edges coated yellowish-brown. (The back part of the tongue is black.) Therefore coldness of the tongue relates to Veratrum alb., to Camphor; but Camphor has not the blackness. You will see this condition of affairs in low forms of fever, after a violent diarrhœa, with sweating and coldness.

Under sixteen you see hiccough after hot drinks, and also aggravation from hot drinks. Under desires and aversions you find there a part of the red string: craves fruits, juicy food, like Phos. acid and China; voracious appetite. Hunger and appetite between the paroxysms of vomiting. That is like Æthusa. You will want to know how to distinguish Veratrum alb. from Æthusa in relation to that symptom. You will find that in Æthusa the baby will nurse itself full, leave the breast and vomit it up; it will vomit the milk and vomit the contents of the stomach; the first that comes up is something that looks like "smear-case," but that which has gone into the stomach last will appear just as it was taken. It will vomit curdled milk and uncurdled milk, and lie down and go to sleep and wake up as hungry as a wolf. Veratrum alb. will vomit profuse, watery matter first, and mixed with water and bile afterward. Perhaps there may be a diarrhœa. She will even, after vomiting, be as hungry as a wolf, and eat almost anything you give her to fill up her stomach. In a few minutes it comes up again. She will want cold things, and in a little while it comes up as before. Although there have been considerable solids eaten, she will vomit a great amount of liquid also, for it seems perfectly in keeping with Veratrum alb. for liquids to be hurled into the stomach and intestines, then to be poured away by vomiting and purging. Æthusa has some of the blueness of the skin, the hypocratic countenance and the sinking away about the nose and face—the pointed nose and face; but the cold sweat with which the vomiting is generally associated as well as the purging and the great amount of liquid and watery vomit—that will help you to distinguish it from Veratrum alb. There is aversion to warm things. You must remember these facts in relation to the remedy—warm food and warm drinks. There are two or three other remedies having that. In Phos. and Pulsatilla warm things taken into the stomach will be vomited as in Veratrum alb. Nausea, with sensation of fainting, generally with violent thirst. (That is an important symptom.) Now, observe how the characteristic feature runs through the vomit: vomiting with continuous nausea and great prostration. Thin, blackish, or yellowish substance, of bile and blood, are the characteristics. You may have that with many other remedies. Bnt you must remember that there is profuse sweating, thirst, and exhaustion; the sweat is cold and there is likely to be a diarrhœa. This is the great sphere for which Veratrum alb. has been so useful; vomiting, diarrhœa, and sweat.

There may be nothing in the stool of Veratrum alb. to guide you to it; but if it is pouring away copiously—if there is copious vomiting, with the cold sweat, you wouldn't have to hesitate long before selecting the remedy. The pains in the stomach are likely to be very violent and may be associated with cramps in the extremities. Great retching; gastric catarrh; great weakness; cold; sudden sinking; vomiting of blood with coldness; fainting fits; cold sweat, nausea, and vomiting. We have coldness in the abdomen, and also great burning in the abdomen. There is a tympanitic condition of the abdomen with great sensitiveness to the touch, with burning or coldness. Chronic coldness of the abdomen is a very common sensation with pregnant women and in women suffering from uterine disturbances. Coldness in the abdomen. You will very often in that case think of Veratrum alb. Always think of it when you have marked coldness of the abdomen, peritonitis, etc., vomiting of fæcal matter from spasms of the bowels, and he is covered with cold sweat. Here you have the symptoms that lead to Veratrum alb. in Asiatic cholera; stools watery, greenish, mixed with flakes; gushing, profuse, rice-water discharges, with tonic cramps commencing in the hands and feet, spreading all over; sunken, hypocratic face. Asiatic cholera: Watery, inodorous, gushing, flaky, thin, papescent, mucous stools. Now, Arsenicum differs from this remedy, as I said, from the fact that it is scanty in its stools; it has the prostration of Veratrum alb., if anything more marked, if that were possible; it has a sweat, but it is not cold, as Veratrum alb. is cold. Arsenicum has great coldness, but still there is not the icy coldness of Veratrum alb.

Nearly all the cholera remedies have more or less of cramping. Veratrum alb. has cramps in the soles, in the calves particularly, and in the fingers and toes. Cuprum is marked for the cramps, Camphor for its coldness, and Veratrum alb. for its cold sweat. Now, these things predominate, yet they all have these things in a general way—they have these things in common, but when the coldness is extreme, and not much sweat, but cold and dry, it is Camphor. Coldness, dryness, and blueness is Camphor. The terrible cramps that come on in the abdomen that make him screech and scream with the pain, the cramps extending to the chest particularly, that calls for Cuprum. Cuprum has the vomiting and purging nearly as great as Veratrum alb., but it has not the cold sweat in any marked degree; it has exhaustion, like Veratrum alb.; but it has not the cold sweat. It is marked for the collapse. Veratrum alb. for the cold sweat. Camphor for the coldness.

There are cases of dry cholera where the patient is taken down simply with coldness; no vomiting, no purging; it seems that he has not the vitality to vomit and purge. Neither does he sweat. He is simply cold and blue, almost unconscious, going right into collapse. These cases die without Camphor, and Camphor is the only remedy that we have that can correspond to that state. Your patient's life depends upon Camphor. You can do with Camphor all that you can expect to do because we have no other remedy for that state of affairs. Where the cold sweat, and profuse vomit and purging, and watery character of the diarrhœa predominate, you have Veratrum alb. Where the cramps predominate you may have Cuprum. Arsenicum has more or less of the coldness; it has more of the restlessness in the beginning than any remedy, and the common feature is for Arsenicum cholera to commence after midnight. The Veratrum alb. case may commence any time, no matter when, because its distinctive feature is that it has no time. Arsenicum has also its own characteristic.

The Secale cholera I presume you could picture to yourselves; but owing to the fact that Veratrum alb. is sometimes made better by a little cold—cold things—and that Camphor particularly is made better in its coldness by uncovering, you will have to distinguish it from Secale.

